# Disentangling the Tissue-Specific Variations of Volatile Flavor Profiles of the *Lentinula edodes* Fruiting Body

**DOI:** 10.3390/foods13010086

**Published:** 2023-12-26

**Authors:** Yuan Guo, Jing Zhao, Huixian Wei, Qi Gao, Shuang Song, Yangyang Fan, Dong Yan, Yu Liu, Shouxian Wang

**Affiliations:** 1 Beijing Engineering Research Center for Edible Mushroom, Institute of Plant Protection, Beijing Academy of Agriculture and Forestry Sciences, Beijing 100097, China; guoyuan@ippbaafs.cn (Y.G.); gaoqi@ippbaafs.cn (Q.G.); songshuang@ippbaafs.cn (S.S.); fanyangyang@ippbaafs.cn (Y.F.); yandong@ippbaafs.cn (D.Y.); ly6828@sina.com (Y.L.); 2 College of Horticulture and Plant Protection, Inner Mongolia Agricultural University, Hohhot 010018, China; zj09171215@163.com; 3 College of Agriculture and Food Engineering, Baise University, Baise 533000, China

**Keywords:** *Lentinula edodes*, volatile flavor, metabolomics, GC–MS, tissue specificity, multivariate analysis

## Abstract

For *Lentinula edodes*, its characteristic flavor is the key determinant for consumer preferences. However, the tissue-specific volatile flavor variations of the fruiting body have been overlooked. Here, we comprehensively investigated the volatile flavor profiles of different tissues, including the pileus skin, context, gill, and stipe of the fruiting body, of two widely cultivated *L. edodes* strains (T2 and 0912) using the gas chromatography–mass spectrometry (GC–MS) technique combined with a multivariate analysis. We show that the eight-carbon and sulfur compounds, which represented 43.2–78.0% and 1.4–42.9% of the total volatile emissions for strains 0912 and T2, respectively, dominated their volatile profiles. Compared with strain T2, strain 0912 had a higher total content of eight-carbon compounds but a lower total content of sulfur compounds in the fruiting body. The sulfur compounds represented 32.2% and 42.9% of the total volatile emissions for strains 0912 and T2, respectively. In contrast, they constituted only 1.4% in the stipes of strain 0912 and 9.0% in the skin of strain T2. The proportions of the predominant C8 compounds (1-octen-3-one, 1-octen-3-ol, and 3-octanone) and sulfur compounds (lenthionine, 1,2,4-trithiolane, dimethyl disulfide, and dimethyl trisulfide) changed depending on the tissues and strains. Using machine learning, we show that the prediction accuracy for different strains and tissues using their volatile profiles could reach 100% based on the highly diverse strain- and tissue-derived volatile variations. Our results reveal and highlight for the first time the comprehensive tissue-specific volatile flavor variations of the *L. edodes* fruiting body. These findings underscore the significance of considering strain and tissue differences as pivotal variables when aiming to develop products with volatile flavor characteristics.

## 1. Introduction

*Lentinula edodes* (Berk.) Pegler, known as “Xianggu” in China and “shiitake” in Japan, is the second most cultivated and most popular edible fungus in the world [1,2]. In China, *L. edodes* is of the greatest importance in the industry of edible fungi, as its annual yield accounts for over one third of the total mushroom production according to the latest production statistics [3]. *L. edodes* can be consumed either for therapeutic purposes or as a nutritional food. It harbors abundant bioactive molecules with proven pharmacological properties, including polysaccharide, lentinan, eritadenine, ergosterol, etc. [4]. These bioactive compounds have been shown to confer it multiple therapeutic capacities for preventing and treating diseases involving cancer, depressed immune function, hyperlipidemia, obesity, etc. [4,5]. *L. edodes* is the first medicinal macrofungus to enter the realm of modern biotechnology. As a food, it is rich in various nutrients such as minerals, vitamins, unsaturated fatty acid, proteins, and dietary fiber [4,6]. It has been cultivated and consumed around the globe, especially in eastern Asia [7].

*L. edodes* possesses a characteristic aroma. The aroma of a fresh *L. edodes* fruiting body is largely eight-carbon (C8) compounds comprising 1-octen-3-ol, 2-octen-1-ol, 1-octen-3-one, 3-octanone, 3-octenol, etc. [8,9,10]. The content of C8 compounds can account for more than 70% of the total volatile emission [10]. Among those C8 compounds, the so-called “mushroom alcohol” 1-octen-3-ol is the main contributor to the aroma of fresh *L. edodes* [11]. C8 compounds are synthesized from the oxidation and cleavage of the fatty acid linoleic acid through the lipoxygenase (LOX) pathway [12,13]. Some of the C8 compounds have been used as flavoring agents in the food industry. The odor of 1-octen-3-ol can be described as sweet and earthy, reminiscent of lavender–lavandin, rose, and hay. It has been proven to be an important contributor to meat flavor and is therefore used to enhance chicken flavors [14,15]. 3-octanone, with an odor of butter, herb, and mold, is positively correlated with cooked meat [16,17].

The C8 compounds, however, can describe many fresh mushrooms [18,19,20]. Thus, it cannot be treated as the key characteristic distinguishing it from other mushrooms. The typical aroma of dry *L. edodes* can be characterized by a blend of cyclic and straight-chain sulfur-containing compounds, including lenthionine, 1,2,4-trithiolane, 1,2,4,5-tetrathiane, dimethyl disulfide, dimethyl trisulfide, etc. [8,9,21,22]. The content of sulfur flavor compounds can account for 22.91% to 74.74% of the total content of volatile emissions in dried shiitake [23] and 19.41–21.53% of the total content in fresh shiitake [24,25]. The synthesis of sulfur flavor compounds involves both enzymatic and nonenzymatic reactions. The biosynthesis of volatile sulfur compounds starts from the precursor lentinic acid, followed by a two-step enzyme-catalyzed reaction: The γ-glutamyl peptide bond of lentinic acid is hydrolyzed by γ-glutamyl transpeptidase (GTT) and releases γ-glutamylpeptides. Then, γ-glutamylpeptides are hydrolyzed by cysteine sulfoxide lyase (C-S lyase) and produce thiosulfate. The thiosulfate is thereafter subjected to a complex nonenzymatic reaction through which various sulfur-containing flavor compounds, including lenthionine, are formed [24,26]. The characteristic aroma, which is conferred by the sulfur-containing compounds, is one of the key factors determining the quality of *L. edodes* [27,28,29]. The sulfur-containing compounds can function as both flavor enhancers in food [30] and as medicines to treat microbial infections, cancer, and platelet aggregation [4].

The analysis of mushroom flavor compounds involves two steps, including extraction, identification, and quantification. The former can be achieved via steam distillation (SD), simultaneous distextraction (SDE), solvent-assisted flavor evaporation (SAFE), supercritical fluid extraction (SFE), and solid-phase microextraction (SPME). Among these methods, SPME is the most widely used due to its simplicity, low cost, high sensitivity, speed, and solvent-free and nondestructive processes [19,31]. The latter step includes some of the advanced methods that can achieve fast, real-time, and non-invasive measurements, for example, the electronic noise based on advanced sensors and computer technology, and the extraction-free proton-transfer-reaction time-of-flight mass spectrometer (PTR–TOF–MS) [32]. However, these methods are not well established for the analysis of mushroom volatile flavor compounds. The gas chromatography–mass spectrometry (GC–MS) technique is the most used method for the identification and quantification of mushroom aroma components. GC–MS combines the separation capabilities of GC with the detection properties of MS, enabling efficient, rapid, and convenient analysis of mushroom flavor compounds [19]. 

Agaricomycetes, including *L. edodes*, develop distinct specialized structures, including the pileus skin, context, gill, and stipe of the fruiting body. Plenty of studies have applied the SPME–GC–MS method to analyze the volatile flavors of the fruiting bodies of mushrooms [33,34,35]. It was found that in *Agaricus bisporus*, the cap and gills emitted more 1-octen-3-ol than the stipe, and the cap produced a more desirable fresh and cooked aroma [36]. However, in *Pleurotus ostreatus*, the highest content of 1-octen-3-ol was found in the stipe, followed by the cap and base of the fruiting body [37]. In *L. edodes*, the flavor profiles in different parts of the fruiting body can also be different. Chen et al. [38] reported that the concentrations of most of the C8 compounds and sulfur compounds are markedly higher in the pileus than in the stipe of *L. edodes*. However, the two predominating compounds, 1-octen-3-ol and lenthionine, showed comparable contents in the pileus and stipe [38]. Conversely, another study showed that the content of 1-octen-3-ol in the pileus was significantly lower than that in the stipe of *L. edodes* [39]. Taken together, the volatile flavor profiles can be different depending on the different parts of the fruiting body, though in current studies, the fruiting body is roughly divided into the cap and stipe. Overall, only a limited number of studies have addressed the variations in volatile flavor compounds among different strains and tissues of *L. edodes*. The volatile flavor constitutions of different tissues of the *L. edodes* fruiting body are still poorly understood. In particular, the flavor profiles of the skin and gill of the *L. edodes* fruiting body remain as a gap in knowledge. To decipher the overall and detailed differences in the expression of flavor compounds in different strains and tissues of *L. edodes*, we investigated the volatile flavor profiles of the pileus skin, context, gill, and stipe of the fruiting bodies of two *L. edodes* strains that are widely cultivated in China using the GC–MS technique combined with a multivariate analysis. Our findings demonstrate for the first time the exhaustive tissue-specific volatile flavor variations of the *L. edodes* fruiting body.

## 2. Materials and Methods

### 2.1. Sample Preparation 

The mature fruiting bodies of fresh *L. edodes* of strains 0912 and T2 were selected for experimental use. These two strains are widely cultivated in northern China. Fruiting bodies of *L. edodes* were taken from the well-established base of North Wild Edible Fungus Development Co., Ltd., located in Guangling county, Shaanxi province, China. Different tissues, including the skin of the cap, context, gill, and stipe of the fruiting body, were carefully split up and then cut into thin slices for the following volatile flavor sampling. All fruiting body tissue samples were measured in triplicate. 

### 2.2. Volatile Compound Extraction 

Fresh homogenized samples (ca. 16 g) of different tissues were transferred to glass headspace amber vials (15 mL, Anpel Laboratory Technologies, Shanghai, China). The vials containing samples were then incubated in a water bath at 55 °C for 1 h to enhance volatile emission. Volatile compounds were extracted via the solid-phase microextraction (SPME) method for 1 h using a fused silica fiber (75 μm length) coated with a 50/30 μm layer of divinylbenzene/carboxen/polydimethylsiloxane (DVB/CAR/PDMS) (Supelco^®^ Analytical, Merck KGaA, Darmstadt, Germany). Delta-2-carene were used as an internal standard.

### 2.3. Volatile Compound Measurements with GC–MS

The extracted volatile compounds were analyzed using a GC–MS system (GC type 7890A, MS type 7000C; Agilent Technologies, Palo Alto, CA, USA) equipped with a 5% phenyl-methylpolysiloxane phase capillary column (30 m × 250 μm × 0.25 μm J&W HP–5MS; Agilent Technologies, Palo Alto, CA, USA). Volatile compounds were desorbed from the extraction fiber in the GC injection port at 250 ℃ for 10 min [11]. The GC–MS conditions were configured as follows: carrier gas, 99.999% helium gas; flow rate, 1.0 mL/min; oven temperature program: initial temperature of 40 °C, maintained for 1 min, and heating at rate of 10 ℃/min to 250 °C, which was maintained for 1 min; ionization mode, EI; ion source temperature, 230 °C; quadrupole temperature, 150 °C; mass scan range, 35–550 u. Non-isothermal Kovats retention indices were calculated according to generally accepted standards [40] based on the chromatography retention times of a saturated alkane mixture (C7–C30; Anpel Laboratory Technologies Inc., Shanghai, China). Annotations of peaks were performed by comparing the mass spectra against libraries of reference spectra (NIST 11, Wiley 275) and non-isothermal Kovats retention indices found in the literature [41,42]. Quantifications of volatile compounds were performed following the well-established method [11,41,43]. The GC–MS data were normalized to the fresh weight of the fruiting body used for the volatile sampling.

### 2.4. Statistics

To analyze the tissue-dependent volatile profile patterns, orthogonal projections to latent structures discriminant analysis (OPLS–DA) was performed using SIMCA–P software (Version 14.1, Umetrics, Umeå, Sweden). Otherwise, all data analysis and visualization were performed in the R Studio version 2023.6.1.524 integrated development environment (IDE) [44] using R version 4.3.1 (R Core Team, 2018) [45]. A two-way analysis of variance (ANOVA) was performed to test the significance (Duncan’s post hoc test, *p* < 0.05) of the volatile emissions between different sample groups. The pairwise Spearman correlations and corresponding significance values (*p*-values) between compounds were calculated using the “Hmisc” package. The significant correlations (*p* < 0.05) were selected for a further network analysis. The correlation matrix was constructed and visualized using the “corrplot” package [46]. The highly interconnected communities were computed using the “ggraph” package [47] with the Fruchterman–Reingold layout algorithm [48] from the “igraph” package [49]. The Random Forest–Recursive Feature Elimination (RF–RFE) algorithm [50,51,52] was used to identify the volatile biomarkers of different strains and tissues of *L. edodes*. The number of variables randomly sampled as candidates at each split (mtry) and the number of trees to grow (ntree) were tuned (grid search) using the “caret” package to obtain the optimal predictive ability and accuracy [53]. The sizes of the volatile biomarkers were determined with a prediction accuracy exceeding 80% and a Cohen’s kappa value above 0.7, ensuring reliable prediction performance [54].

## 3. Results

### 3.1. Overall Volatile Emissions from Different Tissues of L. edodes Fruiting Body

**Figure 1:**
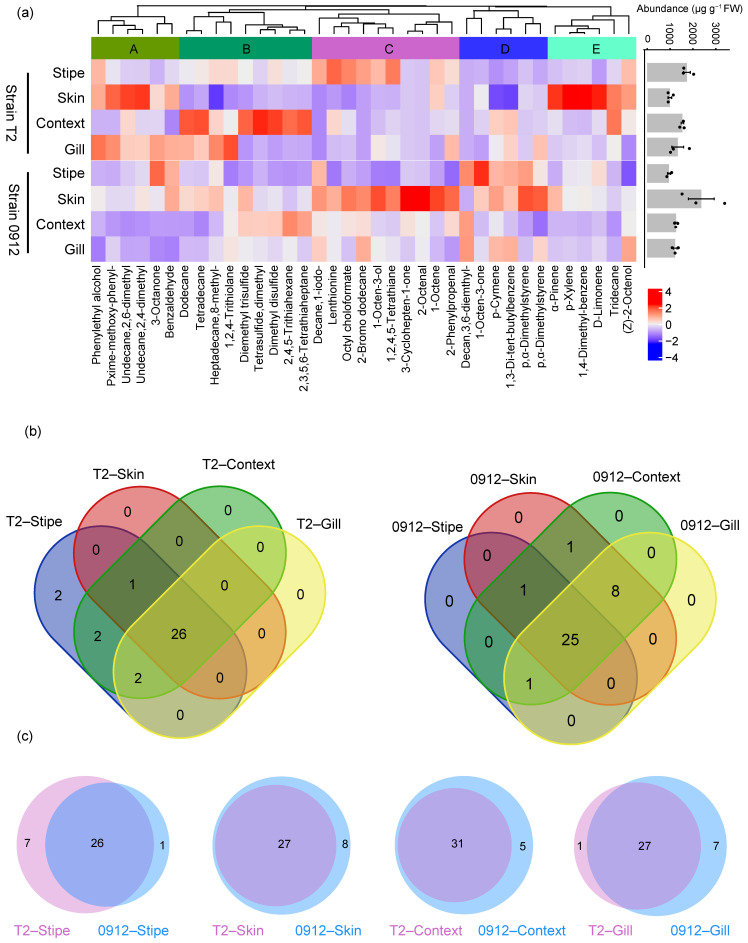
Overall volatile emissions from different tissues of fruiting bodies from different strains of *L. edodes.* (**a**) Heatmap visualizing the volatile profiles and the corresponding total abundance values of different samples (μg g^−1^ of FW). (**b**) Venn diagrams depicting the volatile profiles of the different tissues of strains T2 and 0912. (**c**) Venn diagrams describing the same tissues between different *L. edodes* strains. Numbers in the Venn diagrams show the number of shared or unique compounds. FW indicates fresh weight.

Overall, 33 and 36 volatile compounds were detected from different tissues of strain T2 and strain 0912 fruiting bodies, respectively (Table 1 and Table S1). For strain T2, the highest content of volatile emissions was observed in the stipe and the lowest was observed in the skin, whereas the opposite trend was observed for strain 0912. For both strains, total emission levels were comparable in the context and gill (Table S2 and Figure 1). Using hierarchical clustering, we showed that the detected compounds could be gathered into five groups (A, B, C, D, and E). Some of the subgroups demonstrated clear strain- or tissue-dependent chemotypes. For example, the compounds in group A were generally abundant in strain T2, and the compounds in groups C and D were abundant in strain 0912. Group B, which predominantly consists of sulfur compounds, was abundant in this context, particularly for strain T2. Group E, which largely contains terpenes and benzenoids, was characteristic of the skin of strain T2. A Venn analysis revealed that most of the compounds were shared among different tissues for both strains, although their abundances exhibited pronounced variations. For strain T2, two compounds, i.e., decane,1-iodo- and 2-bromo dodecane, were found to be unique to the stipe. Two typical acyclic sulfur flavor compounds, dimethyl trisulfide and tetrasulfide dimethyl, were only detected in the stipe and context (Figure 1b). The same tissues of different strains shared most of their compounds. For the stipe, one compound (decan,3,6-diemthyl-) was found to be unique to strain 0912, and seven compounds were found to be unique to strain T2 (dimethyl trisulfide, tetrasulfide, dimethyl, lenthionine, 2,3,5,6-tetrathiaheptane, 2,4,5-trithiahexane, 1-octene, and 2-bromo dodecane). There were no compounds unique to the skin of strain T2. For the context, five compounds (decane,1-iodo-, cyclohepten-1-one, decan,3,6-diemthyl-, 2-octenal, and 2-bromo dodecane) were only detected in strain 0912, whereas no compounds were unique to the context of strain T2. The gill of strain 0912 had seven unique compounds (dimethyl trisulfide, decane,1-iodo-, tetrasulfide, dimethyl, decan,3,6-diemthyl-, 2-octenal, 1-octene, and 2-bromo dodecane), whereas strain T2 had only one unique compound (phenylethyl alcohol) (Figure 1c and Table S3).

### 3.2. Chemical Diversity of the Volatile Compounds in Different Tissues of L. edodes Fruiting Body

**Figure 2:**
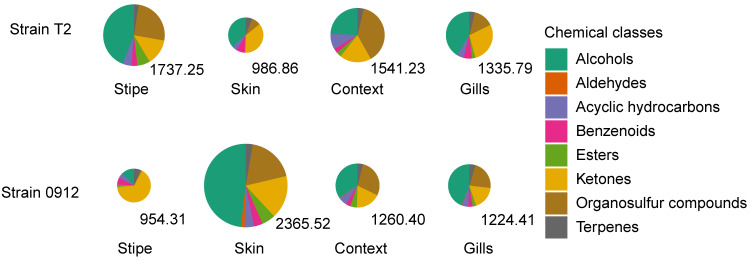
Venn diagrams demonstrating the chemical diversity of the volatile profiles of the different tissues of strains T2 and 0912. Numbers under pies indicate the total abundance of the different compounds (μg g^−1^ of fresh weight).

To illustrate the chemical diversity of the volatile profile, we classified the volatile compounds into eight chemical classes (alcohols, aldehydes, acyclic hydrocarbons, benzenoids, esters, ketones, sulfur compounds, and terpenes). The chemical diversities were shown to be similar across different tissues. For both strains, the volatile profiles were dominated by alcohols, ketones, and sulfur compounds, which accounted for more than 80% of the total volatile emissions. However, in the stipe of strain 0912, only small amounts of sulfur compounds were detected (1.43%). For both strains, most of the sulfur compounds, which provide the typical aroma of a dried fruiting body, were found in the context. For strain T2, the lowest amount of sulfur compounds was detected in the skin, whereas for strain 0912, this value was lowest in the stipe (Figure 2).

### 3.3. The Profiles of C8 and Sulfur Compounds Differed in Different Tissues and Strains

**Figure 3:**
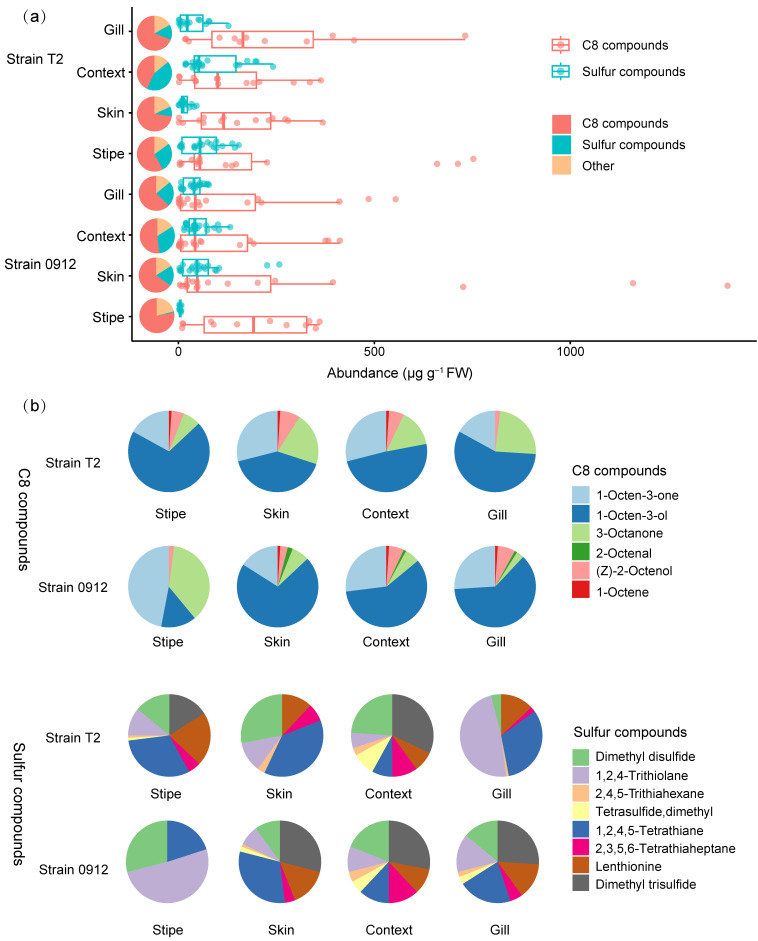
The total abundance (μg g^−1^ of FW) of the eight-carbon (C8) and sulfur compounds in different tissues of strains 0912 and T2 (**a**). Venn diagrams (**b**) demonstrating the compositions of individual C8 and sulfur compounds in different tissues of strains 0912 and T2. FW indicates fresh weight.

The C8 and sulfur compounds, which characterize the aromas of fresh and dried *L. edodes* fruiting bodies, respectively, dominated the volatile profiles of different tissues in the two strains. The C8 compounds accounted for 43–78% of the total volatile emissions in different tissues of the two strains, followed by sulfur compounds, which represented 19–43%. However, the stipe of strain 0912 contained only 1.4% sulfur compounds (Figure 3a). For strain T2, the highest content of C8 compounds was found in the stipe, followed by the gill, skin, and context. In contrast, for strain 0912, the highest content was in the skin, followed by the gill, stipe, and context. For strain T2, the sulfur compounds were highest in the context, followed by the stipe, gill, and skin. However, they were highest in the skin, followed by the context, gill, and stipe in strain 0912 (Figure 3a).

There are six C8 compounds and eight sulfur compounds that were detected in all samples. For strain 0912, the stipe had the lowest diversity of C8 compounds. The mushroom alcohol, 1-octen-3-ol, was predominant in all tissues of both strains, except for the stipe of strain 0912. Additionally, two ketone compounds, 1-octen-3-one and 3-octanone, significantly contributed to the C8 profiles in different tissues of the two strains. These three C8 compounds accounted for 91–98% of the total C8 content (Figure 3b). Similar to the trend observed for C8 compounds, the stipe of strain 0912 exhibited the lowest diversity of sulfur compounds. The most important sulfur flavor compound, lenthionine, was not detected in the stipe of strain 0912, whereas it constituted the largest proportion of the total sulfur compounds in the stipe of strain T2. Surprisingly, the ratio of lenthionine was consistently lower in the context compared to other tissues. Except for lenthionine, the two straight-chain sulfur compounds dimethyl disulfide and dimethyl trisulfide, together with the cyclic sulfur compound 1,2,4-trithiolane, dominated the sulfur flavor profiles across different tissues for both strains, though their ratio varied depending on the tissue and strain (Figure 3b).

### 3.4. Correlations of C8 and Sulfur Compounds

**Figure 4:**
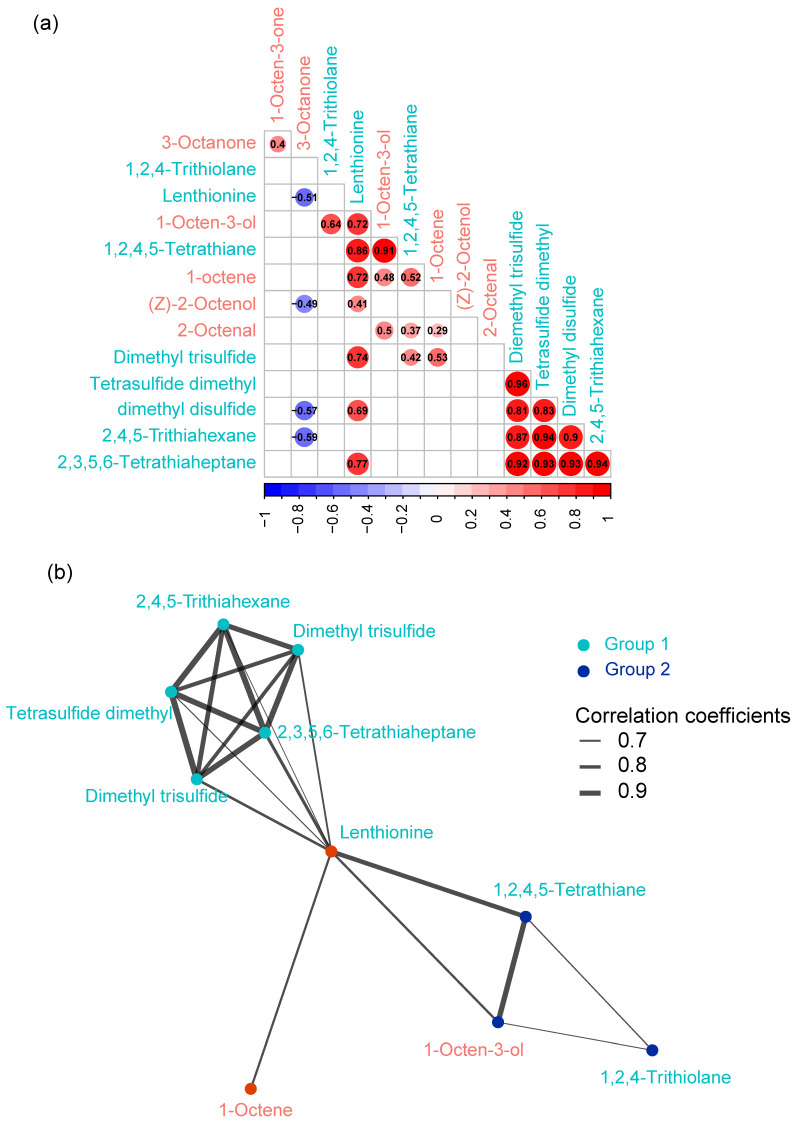
Correlations of eight-carbon (C8) and sulfur compounds detected in different samples. (**a**) Correlation matrix demonstrating the significant correlations (*p* < 0.05) of different C8 and sulfur compounds. Numbers indicate the correlation coefficients. (**b**) Correlation network showing the sub-communities detected with k-means clustering method.

To infer relationships between C8 and sulfur compounds, we conducted a Spearman’s rank correlation analysis. The results showed that the significant (*p* < 0.05) paired correlations were largely positive. Only four moderate (0.4 < r < 0.6) negative correlations were found between 3-octanone and lenthionine, Z-2-octenol, dimethyl disulfide, and 2,4,5-trithiahexane (Figure 4a). Further, we performed a community detection analysis to identify the module of highly connected compounds. The results showed that most sulfur flavor compounds were closely connected, with the exception of 1,2,4-trithiolane. Surprisingly, none of the C8 compounds demonstrated connections with one another (Figure 4b).

### 3.5. The Tissue-Dependent Volatile Profile Patterns Predict Different L. edodes Strains and Tissues

**Figure 5:**
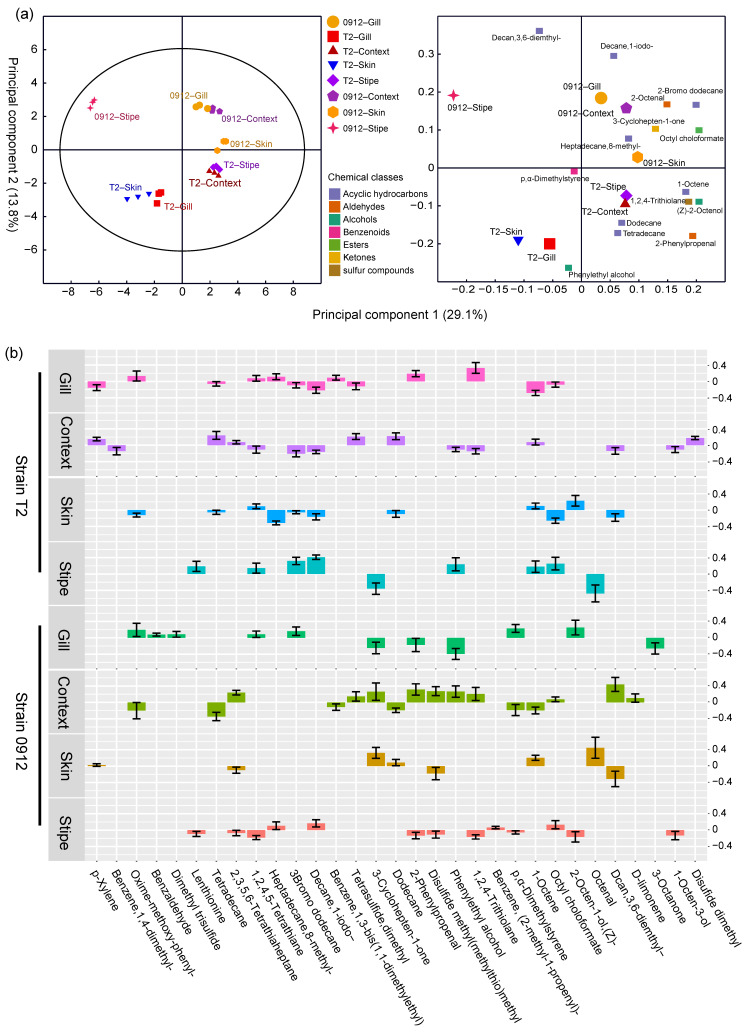
Orthogonal partial least square discriminant analysis (OPLS–DA) of volatile profiles of all samples. (**a**) OPLS–DA score and loading plot showing the patterns of different tissues from different strains based on their volatile profiles. Ellipse: Hotelling’s T2 (95%) regression coefficients related to scaled and centered X variables are displayed. The top compounds based on VIP value are shown in the loading plot. (**b**) Correlation coefficient plots of OPLS–DA showing the relationship between the X and Y variables for the predictive components. The size of the scaled coefficient represents the change in the Y variable when the X variable varies from 0 to 1 in coded units. The error bars indicate the confidence intervals of the coefficients. A coefficient is significant (above the noise) when the confidence interval does not include zero.

The OPLS–DA analysis revealed that the volatile profiles of different tissues and strains were significantly different (CV–ANOVA, *p* < 0.05). The first two principal components explained a total of 42.9% of the variation. Bar plots (Figure 5b) illustrate the significant regression coefficients of each compound for the different tissues. The number of compounds with significant correlations varied depending on the tissue and strain. The skin of strain T2 had the lowest number of significantly correlated compounds, while the numbers for other tissues were comparable. Lenthionine was only positively correlated with the stipe of strain T2, whereas it was negatively correlated with the stipe of strain 0912. 1-octen-3-ol was exclusively negatively correlated with the context in strain T2 and the stipe in strain 0912. Some compounds, such as dimethyl disulfide, dimethyl trisulfide, and 3-octanone, exhibited only one significant correlation (Figure 5b).

### 3.6. Volatile Biomarkers of L. edodes Strains and Tissues

**Figure 6:**
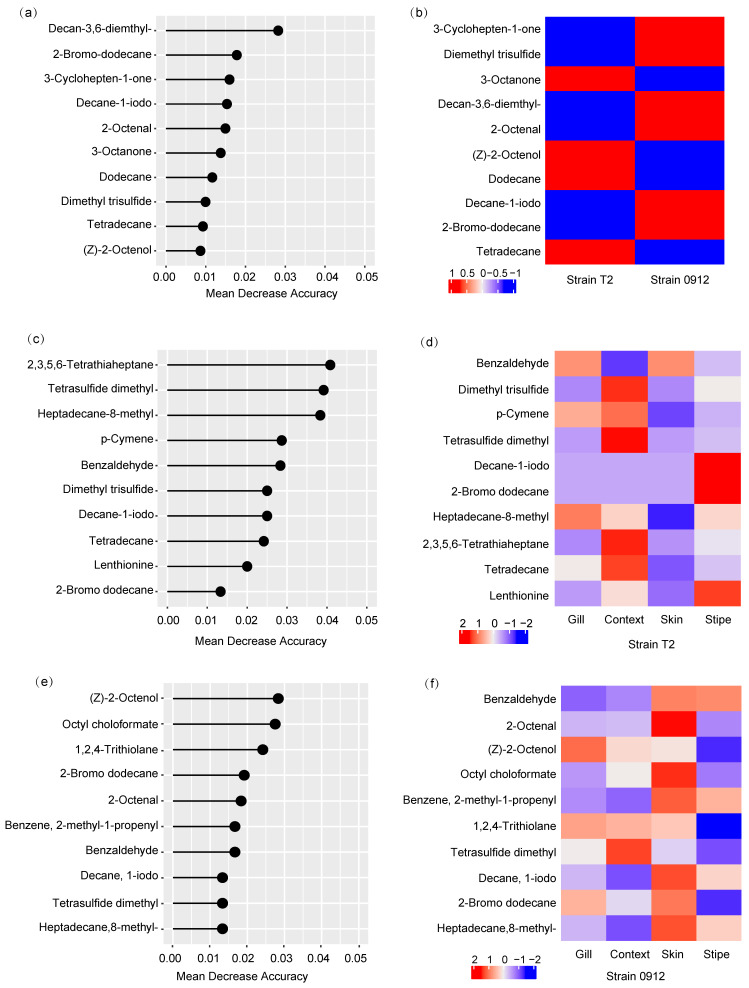
Random forest (RF) analysis showing the top ten compounds discriminating the different strains (**a**) and different tissues of strains T2 (**c**) and 0912 (**e**). Compounds were ranked by the mean decrease in accuracy of the model. Heatmaps were used to visualize the distribution of all individual predictors for prediction of different strains (**b**) and different tissues of strains T2 (**d**) and 0912 (**f**).

As the volatile profiles could characterize the strains and tissues of *L. edodes*, we used a random forest algorithm to identify the corresponding volatile biomarkers. For the group of strains T2 and 0912, the random forest model achieved a prediction accuracy of 100% (*p* < 0.05, Table 1). The top 10 compounds (decan-3,6-diemthyl-, 2-bromo-dodecane, 3-cyclohepten-1-one, decane-1-iodo, 2-octenal, 3-octanone, dodecane, dimethyl trisulfide, tetradecane, and (Z)-2-octenol) resulted in a model accuracy of 88.1% (kappa = 0.71). These biomarkers could be used to distinguish between strains T2 and 0912 (Figure 6). To predict the different tissues of strain T2, their volatile profiles resulted in an overall prediction accuracy of 100% (*p* < 0.01). The prediction accuracy of the stipe, skin, context, and gill using their volatile profiles was also 100% (sensitivity = 1, specificity = 1). The top 14 volatile biomarkers (2,3,5,6-tetrathiaheptane, tetrasulfide dimethyl, heptadecane, 8-methyl-, p-cymene, benzaldehyde, dimethyl trisulfide, decane, 1-iodo, tetradecane, lenthionine, 2-bromo dodecane, (Z)-2-octenol, 2,4,5-trithiahexane, 1,2,4,5-tetrathiane, and octyl choloformate) resulted in a prediction accuracy of 90% (kappa = 0.78). For strain 0912, the overall model accuracy was relatively low (75%, *p*-value = 0.05, kappa = 0.67) due to the low prediction ability for the skin using its volatile profile (balanced accuracy = 0.50, sensitivity = 0). Nevertheless, some compounds still exhibited prediction capacities, such as tetrasulfide dimethyl in the context (Figure 6).

## 4. Discussion

Mushrooms are popular due to their medical value, balanced nutrition, texture, and aroma [31]. Of these characteristics, aroma stands out as the key determinant of consumer preference, especially for mushrooms like *L. edodes*, which has a relatively strong odor [27,31]. In this study, a total of 36 volatile flavor compounds were identified, which differs from previous reports. Qin et al. [55], reported a total of 62 volatile compounds and Xie et al. [56] documented 20 compounds in the fresh *L. edodes* fruiting body. These discrepancies may arise from various experimental factors, such as the specific strains used, the cultivation substrate, the development stage, and the analytical techniques employed [11,31,57]. Nevertheless, current studies have shown that the key volatile flavor compounds, especially the C8 and sulfur compounds, can be maintained independent of experimental variables, although their abundances do change.

For *L. edodes*, numerous studies have investigated the overall volatile profiles of its fruiting body under various cultivation conditions, developmental stages, and drying methods [11,23,24,55,58,59,60,61]. These findings provide an overall understanding of its unique odor. However, the volatile flavor variation in different tissues of the fruiting body remains largely overlooked. In our work, we showed that the stipe of strain T2 and the skin of strain 0912, rather than the context, had the highest volatile emissions per unit weight. Nevertheless, volatiles from the context may contribute the largest part of the total content of the volatile emission, given that the context can represent over 85% of the total fresh weight of the fruiting body [62]. In both strains, the C8 compounds consistently dominated the total volatile profiles of the fresh fruiting body, aligning with findings from other studies [10,11]. More specifically, few compounds, including 1-octen-3-ol, 1-octen-3-one, and 3-octanone, governed the odor of the fresh *L. edodes* fruiting body. These three C8 compounds are commonly predominant in many other mushrooms, for example, *Pleurotus salmoneostramineus* and *Pleurotus sajor-caju* [63], *A. bisporus* [64], and truffles [65]. The individual C8 compounds showed interesting variations depending on the tissues and strains. For example, the mushroom alcohol 1-octen-3-ol was found to be the most predominant compound in the stipe of strain T2, whereas it was the weakest contributor to the C8 profile in the stipe of strain 0912. Such tissue-driven variations are also present in other mushrooms. For instance, in *A. bisporus*, the cap and gill emitted more mushroom alcohol than the stipe. However, in *P. ostreatus*, the stipe was found to be the strongest emitter [36,37].

The volatile profiles of stipes from different strains exhibited marked differences. The sulfur flavor compounds were rather low (1.4%) in the stipe of strain 0912, whereas they were high in the context of strains T2 and 0912. However, Chen et al. [25] reported that the contents of the key flavor compounds, such as thioether and sulfur-containing heterocyclic compounds, were comparable in the stipes and caps of the studied *L. edodes* strain. The abundance of individual sulfur flavor compounds varied greatly in different tissues. One of the most important sulfur flavor compounds, lenthionine, was absent in the stipe of strain 0912, whereas it represented one of the main contributors to the sulfur flavor profile of strain T2. However, in another study, its content was comparable in the pileus and stipes [38]. The proportion of another key sulfur flavor compound, 1,2,4-trithiolane, also changed remarkably depending on different tissues. These findings suggest that strain and tissue differences should be taken into consideration when recycling the stipe for the production of flavoring agents.

Though some studies have documented differences in volatile profiles among different tissues of the *L. edodes* fruiting body [38,39], our data showed for the first time the clear patterns of the volatile profiles for different tissues and strains. Using a random forest algorithm, we found that the volatile profile could predict strains 0912 and T2 with 100% accuracy. Similarly, the prediction accuracy for different tissues using the volatile profile of the studied strains was also 100%. These findings further confirm and highlight the strain- and tissue-specific variations in the volatile emissions of the *L. edodes* fruiting body.

## 5. Conclusions

In conclusion, we demonstrate comprehensive variations in volatile flavors across different strains and tissues. To the best of our knowledge, this work demonstrates the first in-depth investigation into the tissue-specific aroma differences in the *L. edodes* fruiting body. Though the volatile flavor constitution can change depending on many factors such as strains, growth conditions, etc., the key flavor compounds, including C8 and sulfur-containing compounds, can be retained independent of those variables. Given that deep processing based on the flavor and bioactive molecules of *L. edodes* is on the rise, our findings indicate the importance of considering strain and tissue variations to enhance the added value and production efficiency of *L. edodes*-derived products.

## Figures and Tables

**Table 1:** Random forest (RF) model performance of volatile-profile-based models predicting strains T2 and 0912 and their different fruiting body tissues.

Models	Accuracy (%)	Sensitivity (%)	Specificity (%)	Accuracy *p*-Value	Cohen’s Kappa (%)
Strain T2 vs. 0912	100	100	100	0.016	100
Different tissues of strain T2	100			0.004	100
Stipe	100	100	100		
Skin	100	100	100		
Context	100	100	100		
Gill	100	100	100		
Different tissues of strain 0912				0.050	67
Stipe	100	100	100		
Skin	50	0	100		
Context	100	100	100		
Gill	83	100	67		

## Data Availability

Data is contained within the article. The raw GC–MS data of this study are available at the National Genomics Data Center, China National Center for Bioinformation, under PRJCA020932.

## Supplementary Materials

The following supporting information can be downloaded at https://www.mdpi.com/article/10.3390/foods13010086/s1, Table S1. Annotations of all the detected compounds from different tissues of fruiting bodies from the strains T2 and 0912. Table S2. Abundance of volatile compounds detected in different tissues of the fruiting bodies of different *Lentinula edodes* strains. Table S3. The shared and specific compounds of different strains and tissues of the Venn analysis.
