# Supplementary material for: Disentangling the Tissue-Specific Variations of Volatile Flavor Profiles of the Lentinula edodes Fruiting Body
Source: Foods. 2023 Dec 26;13(1):86. doi: 10.3390/foods13010086 (PMC10778291; doi:10.3390/foods13010086)
Supplement: Supplementary file 1 [file foods-13-00086-s001.zip › Table S1_Annotations of all the detected compounds from different tissues of the strain T2 and 0912 fruiting body.pdf]

Table S1. Annotations of all the detected compounds from different tissues of the strain T2 and 0912 fruiting body. RT, Retention time; RI, Kovats retention index.

Number	Compounds	CAS Registry Number	RT	RI	Chemical Class
1	Dimethyl, disulfide	624-92-0	3.339	740	Sulfur compounds
2	1-Octene	111-66-0	3.915	792	Alkenes
3	3-Cyclohepten-1-one	1121-64-8	4.461	820	Ketones
4	p-Xylene	106-42-3	5.14	875	Benzenoids
5	Oxime-methoxy-phenyl-	67160-14-9	6.065	896	Benzenoids
6	α-Pinene	7785-70-8	6.19	928	Terpenes
7	Benzaldehyde	100-52-7	6.654	960	Benzenoids
8	Diemethyl trisulfide	3658-80-8	6.818	975	Sulfur compounds
9	1-Octen-3-one	4312-99-6	6.939	979	Ketones
10	1-Octen-3-ol	3391-86-4	7.089	982	Alcohols
11	3-Octanone	106-68-3	7.194	990	Ketones
12	p-Cymene	99-87-6	7.709	1025	Terpenes
13	D-Limonene	5989-27-5	7.77	1032	Terpenes
14	Decane,3,6-diemthyl-	17312-53-7	8.17	1130	Alkanes
15	2-Octenal	2363-89-5	8.196	1060	Aldehydes
16	(Z)-2-Octenol	26001-58-1	8.37	1071	Alcohols
17	Octyl choloformate	7452-59-7	8.412	1250	Esters
18	α,p-Dimethylstyrene	1195-32-0	8.622	1120	Benzenoids
19	Benzene, (2-methyl-1-propenyl)-	768-49-0	8.734	1218	Terpenes

20	1,2,4-Trithiolane	289-16-7	8.852	1110	Sulfur compounds
21	Phenylethyl alcohol	60-12-8	9.115	1126	Alcohols
22	2,4,5-Trithiahexane	42474-44-2	9.344	1145	Sulfur compounds
23	2-Phenylpropenal	4432-63-7	9.786	1161	Benzenoids
24	Dodecane	112-40-3	10.35	1221	Alkanes
25	Undecane,2,4-dimethyl	17312-80-0	10.51	1223	Alkanes
26	Undecane,2,6-dimethyl	17301-23-4	10.55	1216	Alkanes
27	Tetrasulfide dimethyl	5756-24-1	10.75	1215	Sulfur compounds
28	1,3-Di-tert-butylbenzene	1014-60-4	11.21	1240	Benzenoids
29	Decane,1-iodo-	2050-77-3	11.28	1400	Alkanes
30	2-Bromo dodecane	13187-99-0	11.44	1540	Alkanes
31	Heptadecane,8-methyl-	13287-23-5	11.52	1290	Alkanes
32	Tridecane	629-50-5	11.79	1310	Alkanes
33	1,2,4,5-Tetrathiane	291-22-5	12.67	1332	Sulfur compounds
34	2,3,5,6-Tetrathiaheptane		12.72	1344	Sulfur compounds
35	Tetradecane	629-59-4	13.18	1370	Alkanes
36	Lenthionine	292-46-6	16.45	1622	Sulfur compounds
