# Supplementary material for: Disentangling the Tissue-Specific Variations of Volatile Flavor Profiles of the Lentinula edodes Fruiting Body
Source: Foods. 2023 Dec 26;13(1):86. doi: 10.3390/foods13010086 (PMC10778291; doi:10.3390/foods13010086)
Supplement: Supplementary file 1 [file foods-13-00086-s001.zip › Table S2_Abundance of volatile compounds detected from different tissues of the fruiting body of different Lentinula edodes strains.pdf]

Table S2. Abundance of volatile compounds detected from different tissues of the fruiting body of different *Lentinula edodes* strains. Data shown as mean ± SEM, n = 3; nd = not detected. Different letters indicate significant difference at a significant level of 0.05.

Compounds	Strain 0912				Strain T2			
	Stipe ($\mu g\ g^{-1}$)	Skin ($\mu g\ g^{-1}$)	Context ($\mu g\ g^{-1}$)	Gill ($\mu g\ g^{-1}$)	Stipe ($\mu g\ g^{-1}$)	Skin ($\mu g\ g^{-1}$)	Context ($\mu g\ g^{-1}$)	Gill ($\mu g\ g^{-1}$)
**Alcohols**								
1-Octen-3-ol	107.44 ± 21.17 d	1096.02 ± 197.34 a	389.91 ± 11.24 c	483.50 ± 41.40 bc	708.63 ± 26.82 b	293.61± 38.49 cd	331.03 ± 20.24 cd	524.67 ± 104.79 bc
(Z)-2-Octenol	10.75 ± 0.71 c	39.37 ± 8.87 b	41.03 ± 1.90 b	58.18 ± 6.23 a	52.40 ± 2.03 ab	60.53± 3.93 a	42.42 ± 1.75 b	20.82 ± 2.84 c
Phenylethyl alcohol	1.65 ± 0.35 bc	3.69 ± 1.08 abc	1.68 ± 0.06 bc	nd	5.72 ± 0.71 abc	6.14 ± 4.44 ab	1.71 ± 0.19 bc	8.07 ± 2.21 a
**Aldehydes**								
2-Octenal	nd	34.17 ± 7.87 a	5.74 ± 0.24 b	5.00 ± 0.44 b	nd	nd	nd	nd
**Acyclic hydrocarbons**								
Decan,3,6-diemthyl-	4.38 ± 0.11 a	nd	3.90 ± 0.12 a	4.01 ± 0.23 a	nd	nd	nd	nd
Dodecane	4.85 ± 0.24 bc	6.38 ± 1.30 abc	3.71 ± 0.12 bc	4.48 ± 0.21 c	5.59 ± 0.21 abc	3.53 ± 0.18 ab	9.80 ± 0.32 bc	7.53 ± 1.12 a
Undecane,2,4-dimethyl	0.87 ± 0.17 abc	1.04 ± 0.29 ab	0.55 ± 0.03 c	0.64 ± 0.03 bc	0.82 ± 0.07 abc	1.89 ± 1.37	0.86 ± 0.02 abc	1.17 ± 0.17 a
Undecane,2,6-dimethyl	2.68 ± 0.53 abc	2.91 ± 0.64 ab	1.75 ± 0.09 c	2.12 ± 0.10 bc	2.72 ± 0.23 abc	6.02 ± 4.36	3.65 ± 0.10 abc	3.85 ± 0.56 a
Decane,1-iodo-	4.52 ± 0.39 ab	5.65 ± 1.39 a	3.03 ± 0.20 b	3.83 ± 0.15 ab	4.94 ± 0.42 ab	nd	nd	nd
2-Bromo dodecane	nd	1.83 ± 0.54 a	0.93 ± 0.04 a	1.46 ± 0.15 a	1.59 ± 0.14 a	nd	nd	nd
Heptadecane,8-methyl-	10.68 ± 0.95 bc	13.22 ± 3.34 ab	7.02 ± 0.42 c	8.90 ± 0.40 bc	11.59 ± 0.67 bc	nd	11.89 ± 0.39 abc	16.90 ± 2.83 a
Tridecane	6.40 ± 0.37 cd	7.69 ± 3.72	4.40 ± 0.05 d	5.91 ± 0.31 cd	8.19 ± 0.11 bc	17.28 ± 12.54	17.65 ± 1.05 a	9.74 ± 1.56 b
Tetradecane	12.45 ± 0.85 bcd	18.28 ± 5.00 b	7.81 ± 0.08 d	11.49 ± 0.70 bcd	16.07± 0.18 bc	10.18 ± 1.19 cd	28.36 ±1.88 a	18.66 ± 3.15 b
1-Octene	nd	10.49 ± 4.91	1.48 ± 0.19 b	0.94 ± 0.11 b	5.00 ± 1.29 a	4.61 ± 3.33	2.09 ± 0.35 b	nd
**Benzenoids**								
p-Xylene	3.03 ± 0.39 a	3.21 ± 1.49	1.86 ± 0.12 c	2.41 ± 0.11 abc	2.41 ± 0.26 abc	8.46 ± 6.15	2.05 ± 0.04 bc	2.61 ± 0.03 ab
Oxime-methoxy-phenyl-	3.25 ± 1.01 bc	4.61 ± 0.82 b	2.53 ± 0.10 c	4.32 ± 0.41 bc	4.94 ± 0.73 b	10.34 ± 7.49	3.38 ± 0.19 bc	9.03 ± 0.43 a
Benzaldehyde	37.48 ± 4.79 a	38.45 ± 14.21	14.51 ± 1.02 c	10.83 ± 1.18 c	23.22 ± 2.43 b	38.21 ± 13.50	11.40 ± 0.57 c	37.72 ± 10.71
p,α-Dimethylstyrene	10.11 ± 1.04 ab	12.78 ± 3.01 a	7.69 ± 0.36 ab	10.48 ± 0.41 ab	8.67 ± 0.11 ab	9.25 ± 2.00 ab	9.33 ± 0.38 ab	10.86 ± 0.15 ab
2-Phenylpropenal	1.21 ± 0.13 b	12.32 ± 3.67	7.39 ± 0.77 a	3.03 ± 0.56 b	7.27 ± 0.38 a	5.05 ± 2.06 c	5.62 ± 1.08 ab	8.78 ± 1.92 ab
1,3-Di-tert-butylbenzene	9.10 ± 0.58 a	8.65 ± 1.46 ab	7.04 ± 0.48 abc	9.03 ± 0.12 a	6.55 ± 0.22 bc	5.00± 1.15 c	8.62 ± 0.33 ab	8.37 ± 0.16 ab
**Esters**								
Octyl choloformate	22.36 ± 0.89 c	124.67 ± 26.36 a	61.63 ± 0.66 b	32.02 ± 2.22 bc	117.36 ± 11.21 a	nd	45.84 ± 1.39 bc	31.54 ± 7.48 bc
**Ketones**								

3-Cyclohepten-1-one	nd	40.73 ± 23.02 a	9.11 ± 0.94 b	nd	nd	nd	nd	nd
1-Octen-3-one	348.34 ± 7.67 a	240.82 ± 79.82 ab	177.11 ± 10.55 b	196.93 ± 10.70 b	169.94 ± 28.27 b	208.57 ± 48.87 b	190.55 ± 9.30 b	156.254 ± 33.88 b
3-Octanone	278.22 ± 26.41 a	115.35 ± 67.36 ab	44.13 ± 13.39 bc	24.19 ± 6.42 d	67.15 ± 26.91 cd	151.98± 25.57 bc	99.93 ± 0.94 cd	218.85 ± 54.49 ab
**Sulfur compounds**								
Dimethyl disulfide	3.95 ± 0.77 e	46.19 ± 5.55 cd	78.63 ± 6.55 b	39.09 ± 0.61 cd	63.32 ± 17.16 bc	25.00± 10.76 de	159.30 ± 8.53 a	7.21 ± 2.10 e
Dimethyl trisulfide	nd	133.91 ± 61.80 ab	112.02 ± 10.03 b	72.66 ± 3.76 b	75.47 ± 12.12 b	nd	212.39 ± 14.61 a	nd
1,2,4-Trithiolane	6.90 ± 0.34 c	38.59 ± 7.57 b	41.54 ± 0.53 b	44.01 ± 2.42 b	47.98 ± 6.19 b	10.76 ± 1.80 c	39.18 ± 3.19 b	91.95 ± 18.00 a
2,4,5-Trithiahexane	nd	3.715 ± 0.65 cd	16.24 ± 1.33 a	6.75 ± 0.37 b	6.34 ± 1.39 bc	2.57 ± 1.24 d	18.58 ± 0.58 a	1.94 ± 0.38 b
Tetrasulfide dimethyl	nd	8.38 ± 1.35 c	22.21 ± 2.67 b	10.50 ± 1.34 bc	6.95 ± 1.05 c	nd	58.26 ± 7.94 a	nd
1,2,4,5-Tetrathiane	2.80 ± 0.18 c	142.35 ± 42.08 a	48.05 ± 3.49 bc	60.46 ± 5.34 b	145.12 ± 4.68 a	33.86 ± 3.32 bc	52.26 ± 3.86 bc	61.43 ± 8.88 b
2,3,5,6-Tetrathiaheptane	nd	18.88 ± 5.35 c	48.38 ± 3.22 b	12.96 ± 1.26 cd	23.32 ± 3.92 c	6.15 ± 2.35 d	69.76 ± 5.75 a	4.21 ± 0.81 d
Lenthionine	nd	67.84 ± 13.98 b	38.75 ± 2.01 cd	39.14 ± 7.49 cd	95.39 ± 8.65 a	10.94 ± 2.88 e	51.18 ± 5.91 bc	23.53 ± 2.45 de
**Terpenes**								
α-Pinene	0.78 ± 0.15 b	1.75 ± 0.81	0.85 ± 0.02 ab	0.96 ± 0.05 ab	0.93 ± 0.02 ab	2.72 ± 1.98	1.15 ± 0.03 a	0.99 ± 0.03 ab
p-Cymene	36.84 ± 2.74 ab	38.43 ± 7.70 a	32.27 ± 2.51 abc	36.68 ± 0.98 ab	27.34 ± 0.71 bc	23.26 ± 2.31 c	35.10 ± 0.94 ab	32.58 ± 1.41 abc
D-limonene	16.52 ± 1.59 a	14.79 ± 3.11 ab	12.60 ± 0.84a b	13.88 ± 0.53 ab	10.60 ± 0.41 b	26.87 ± 14.07	14.07 ± 0.38 ab	12.67 ± 0.49 ab
Benzene, (2-methyl-1-propenyl)-	6.71 ± 1.28 a	8.34 ± 3.92	2.95 ± 0.23 b	3.65 ± 0.18 b	3.71 ± 0.21 b	4.09 ± 1.42 b	3.83 ± 0.35 b	3.88 ± 0.11 b

The grey value was excluded for significance test as they have two large within group variation. Abundance of compounds were calculated based on fresh weight of samples.
