# Supplementary material for: Disentangling the Tissue-Specific Variations of Volatile Flavor Profiles of the Lentinula edodes Fruiting Body
Source: Foods. 2023 Dec 26;13(1):86. doi: 10.3390/foods13010086 (PMC10778291; doi:10.3390/foods13010086)
Supplement: Supplementary file 1 [file foods-13-00086-s001.zip › Table S3 The shared and specific compounds of different strains and tissues of the Venn analysis.pdf]

Table S3. The shared and specific compounds of different strains and tissues of the Venn analysis

Groups of comparisons	Number of shared and specific compounds	Compounds
Comparisons of different tissues of strains T2		
Shared by all tissue	26	1,2,4,5-Tetrathiane; Oxime-Methoxy-Phenyl-; (Z)-2-Octenol; P-Xylene; Lenthionine; Dodecane; 2,3,5,6-Tetrathiaheptane; D-Limonene; Undecane,2,6-Dimethyl; 1,3-Di-Tert-Butylbenzene; 1-Octen-3-Ol; Tridecane; Phenylethyl Alcohol; P-Cymene; 3-Octanone; 2,4,5-Trithiahexane;A-Pinene; Tetradecane; Benzene, 1-Methyl-4-(1-Methylethenyl)-; Benzaldehyde; 1,2,4-Trithiolane; 2-Phenylpropenal; Undecane,2,4-Dimethyl; Benzene, (2-Methyl-1-Propenyl)-; Dimethyl Disulfide; 1-Octen-3-One
Shared by stipe, skin and context	1	1-Octene
Shared by stipe, gill and context	2	Octyl Choloformate; Heptadecane,8-Methyl-
Shared by stipe and context	2	Diemethyl Trisulfide; Tetrasulfide,Dimethyl
Specific for stipe	2	Decane,1-Iodo-; Bromo Dodecane
Comparisons of different tissues of strains 0912		
Shared by all tissue	25	1,2,4,5-Tetrathiane; Decane,1-Iodo-; Oxime-Methoxy-Phenyl-; (Z)-2-Octenol; P-Xylene; Dodecane; D-Limonene; Undecane,2,6-Dimethyl; 1,3-Di-Tert-Butylbenzene; Octyl Choloformate; 1-Octen-3-Ol; Tridecane; P-Cymene; 3-Octanone; Heptadecane,8-Methyl-;A-Pinene; Tetradecane; Benzene, 1-Methyl-4-(1-Methylethenyl)-; Benzaldehyde; 1,2,4-Trithiolane; 2-Phenylpropenal; Undecane,2,4-Dimethyl; Benzene, (2-Methyl-1-Propenyl)-; Dimethyl Disulfide; 1-Octen-3-One
Shared by stipe, skin and context	1	Phenylethyl Alcohol
Shared by stipe, gill and context	1	Decan,3,6-Diemthyl-

Shared by skin, gill and context	8	Diemethyl Trisulfide; Tetrasulfide,Dimethyl; Lenthionine; 2,3,5,6-Tetrathiaheptane; 2-Octenal; 2,4,5-Trithiahexane; 1-Octene; 2-Bromo Dodecane
Shared by skin and context	1	3-Cyclohepten-1-One
Comparisons of stipes strains T2 and 0912		
Shared by stipes of strain 0912 and T2	26	Dimethyl Disulfide; P-Xylene; Oxime-Methoxy-Phenyl-; A-Pinene; Benzaldehyde; 1-Octen-3-One; 1-Octen-3-Ol; 3-Octanone; P-Cymene; D-Limonene; (Z)-2-Octenol; Octyl Choloformate; Benzene, 1-Methyl-4-(1-Methylethenyl)-; Benzene, (2-Methyl-1-Propenyl)-; 1,2,4-Trithiolane; Phenylethyl Alcohol; 2-Phenylpropenal; Dodecane; Undecane,2,4-Dimethyl; Undecane,2,6-Dimethyl; 1,3-Di-Tert-Butylbenzene; Decane,1-Iodo-; Heptadecane,8-Methyl-; Tridecane; 1,2,4,5-Tetrathiane; Tetradecane
Specific for stipes of strain 0912	1	Decan,3,6-Diemthyl-
Specific for stipes of T2	7	1-Octene; Diemethyl Trisulfide; 2,4,5-Trithiahexane; Tetrasulfide,Dimethyl; 2-Bromo Dodecane; 2,3,5,6-Tetrathiaheptane; Lenthionine
Comparisons of contexts strains T2 and 0912		
Shared by contexts of strain 0912 and T2	31	Dimethyl Disulfide; 1-Octene; P-Xylene; Oxime-Methoxy-Phenyl-; A-Pinene; Benzaldehyde; Diemethyl Trisulfide; 1-Octen-3-One; 1-Octen-3-Ol; 3-Octanone; P-Cymene; D-Limonene; (Z)-2-Octenol; Octyl Choloformate; Benzene, 1-Methyl-4-(1-Methylethenyl)-; Benzene, (2-Methyl-1-Propenyl)-; 1,2,4-Trithiolane; Phenylethyl Alcohol; 2,4,5-Trithiahexane; 2-Phenylpropenal; Dodecane; Undecane,2,4-Dimethyl; Undecane,2,6-Dimethyl; Tetrasulfide,Dimethyl; 1,3-Di-Tert-Butylbenzene; Heptadecane,8-Methyl-; Tridecane; 1,2,4,5-Tetrathiane; 2,3,5,6-Tetrathiaheptane; Tetradecane; Lenthionine
Specific for contexts of strain 0912	5	3-Cyclohepten-1-One; Decan,3,6-Diemthyl-; 2-Octenal; Decane,1-Iodo-; 2-Bromo Dodecane
Specific for contexts of T2	0	
Comparisons of gills strains T2		

and 0912		
Shared by gills of strain 0912 and T2	27	Dimethyl Disulfide; P-Xylene; Oxime-Methoxy-Phenyl-; A-Pinene; Benzaldehyde; 1-Octen-3-One; 1-Octen-3-Ol; 3-Octanone; P-Cymene; D-Limonene; (Z)-2-Octenol; Octyl Choloformate; Benzene, 1-Methyl-4-(1-Methylethenyl)-; Benzene, (2-Methyl-1-Propenyl)-; 1,2,4-Trithiolane; 2,4,5-Trithiahexane; 2-Phenylpropenal; Dodecane; Undecane,2,4-Dimethyl; Undecane,2,6-Dimethyl; 1,3-Di-Tert-Butylbenzene; Heptadecane,8-Methyl-; Tridecane; 1,2,4,5-Tetrathiane; 2,3,5,6-Tetrathiaheptane; Tetradecane; Lenthionine
Specific for gills of strain 0912	7	1-Octene; Diemethyl Trisulfide; Decan,3,6-Diemthyl-; 2-Octenal; Tetrasulfide,Dimethyl; Decane,1-Iodo-; 2-Bromo Dodecane
Specific for gills of T2	1	Phenylethyl Alcohol
Comparisons of skins strains T2 and 0912		
Shared by skins of strain 0912 and T2	27	Dimethyl Disulfide; 1-Octene; P-Xylene; Oxime-Methoxy-Phenyl-; A-Pinene; Benzaldehyde; 1-Octen-3-One; 1-Octen-3-Ol; 3-Octanone; P-Cymene; D-Limonene; (Z)-2-Octenol; Benzene, 1-Methyl-4-(1-Methylethenyl)-; Benzene, (2-Methyl-1-Propenyl)-; 1,2,4-Trithiolane; Phenylethyl Alcohol; 2,4,5-Trithiahexane; 2-Phenylpropenal; Dodecane; Undecane,2,4-Dimethyl; Undecane,2,6-Dimethyl; 1,3-Di-Tert-Butylbenzene; Tridecane; 1,2,4,5-Tetrathiane; 2,3,5,6-Tetrathiaheptane; Tetradecane; Lenthionine
Specific for skins of strain 0912	8	3-Cyclohepten-1-One; Diemethyl Trisulfide; 2-Octenal; Octyl Choloformate; Tetrasulfide,Dimethyl; Decane,1-Iodo-; 2-Bromo Dodecane; Heptadecane,8-Methyl-
Specific for skins of T2	0	
